# Repeat radiation with bevacizumab and minocycline in bevacizumab-refractory high grade gliomas: a prospective phase 1 trial

**DOI:** 10.1007/s11060-020-03551-3

**Published:** 2020-06-06

**Authors:** Adam L. Cohen, Christopher J. Anker, Brett Johnson, Lindsay M. Burt, Dennis C. Shrieve, Karen Salzman, Randy Jensen, Ken Boucher, Howard Colman

**Affiliations:** 1 Division of Medical Oncology, University of Utah School of Medicine, Salt Lake City, UT USA; 2 Huntsman Cancer Institute, Salt Lake City, UT USA; 3 Division of Radiation Oncology, University of Vermont Larner College of Medicine, Burlington, VT USA; 4 Department of Radiation Oncology, University of Utah School of Medicine, Salt Lake City, UT USA; 5 Department of Radiology, University of Utah School of Medicine, Salt Lake City, UT USA; 6 Department of Neurosurgery, University of Utah School of Medicine, Salt Lake City, UT USA; 7 Department of Epidemiology, University of Utah School of Medicine, Salt Lake City, UT USA

**Keywords:** Glioma, Reirradiation, Minocycline, Bevacizumab

## Abstract

**Introduction:**

There are no effective treatments for gliomas after progression on radiation, temozolomide, and bevacizumab. Microglia activation may be involved in radiation resistance and can be inhibited by the brain penetrating antibiotic minocycline. In this phase 1 trial, we examined the safety and effect on survival, symptom burden, and neurocognitive function of reirradiation, minocycline, and bevacizumab.

**Methods:**

The trial used a 3 + 3 design for dose escalation followed by a ten person dose expansion. Patients received reirradiation with dosing based on radiation oncologist judgment, bevacizumab 10 mg/kg IV every two weeks, and oral minocycline twice a day. Symptom burden was measured using MDASI-BT. Neurocognitive function was measured using the COGSTATE battery.

**Results:**

The maximum tolerated dose of minocycline was 400 mg twice a day with no unexpected toxicities. The PFS3 was 64.6%, and median overall survival was 6.4 months. Symptom burden and neurocognitive function did not decline in the interval between treatment completion and tumor progression.

**Conclusions:**

Minocycline 400 mg orally twice a day with bevacizumab and reirradiation is well tolerated by physician and patient reported outcomes in people with gliomas that progress on bevacizumab.

## Introduction

Gliomas are the most frequent brain tumor, accounting for approximately 12–15% of all brain tumors [1]. Standard treatments following maximal safe resection, depending on histology, include radiation, chemotherapies such as temozolomide, CCNU, or PCV, tumor treating fields, and bevacizumab [2–5]. Recurrent gliomas remain a clinical challenge, however. In particular, once high grade gliomas have progressed following radiation, chemotherapy, and bevacizumab, no treatments are known to prolong survival. Indeed, the 6-month progression-free survival (PFS6) in such situations is generally < 10% in published studies [6, 7].

Although historically repeat fractionated radiation for gliomas was considered prohibitively toxic, modern radiation techniques allow for repeat radiation in many cases [8–14]. Several studies have directly assessed fractionated reirradiation with concomitant bevacizumab and have demonstrated its safety [15–18]. Only two groups have published on reirradiation for high grade gliomas after progression on bevacizumab, showing a median survival of 5.4 months [19] and 4.8 months [20]. Moreover, no study has documented quality of life or cognition following repeat radiation in this population.

Studies have identified activation of nuclear factor kappa B (NFκB) as a key signaling factor promoting the mesenchymal subtype and radiation resistance in glioblastoma (GBM) [21, 22]. Activation of NFκB in glioma stem-cell cultures by tumor necrosis factor (TNF) treatment resulted in radiation resistance that can be reversed by blocking NFκB. To test the role of microglia and NFκB activation on treatment resistance in vivo, treatment with minocycline, an inhibitor of microglia activation, led to a reduction of tumor grade and down-regulation of mesenchymal markers in intracranial glioma stem cell xenograft models [22].

Minocycline is a tetracycline-derivative that is FDA approved as an antibiotic. It also has anti-inflammatory properties that are not shared by all members of the tetracycline family. Because of its known ability to cross the blood–brain barrier [23], minocycline has potential as an anti-glioma agent and as a radiation sensitizer for glioma. Minocycline inhibits matrix metalloproteinase expression by microglia, thus reducing glioma invasion and expansion [24]. Minocycline also induces glioma cell death via autophagy and apoptosis [25]. Animals treated with local administration of minocycline to tumor xenografts have improved survival [26].

The present study is a phase 1 trial of the addition of minocycline to fractionated reirradiation and bevacizumab for people with gliomas that have progressed following all standard treatments, including bevacizumab. The trial incorporated serial measurement of symptom burden and cognitive function.

## Materials and methods

### Study design

An open-label, single arm, Phase 1, dose-escalation and dose-expansion clinical trial was conducted with patients enrolled at a single institution. The trial included adults with radiologically proven recurrent, intracranial glioma who had been previously treated with radiation, temozolomide and bevacizumab. Progression must have been documented by Response Assessment in Neuro-Oncology (RANO) criteria [27], and patients must have been at least 6 months from prior radiation. Subjects were required to have adequate hematologic, hepatic, and renal function and a Karnofsky Performance Status (KPS) of at least 50. The protocol did not limit the size of the tumor as long as the radiation oncologist determined reirradiation was feasible. Exclusion criteria included contraindications to bevacizumab, including uncontrolled hypertension, intracranial hemorrhage, recent surgery, or non-healing wounds, and contraindications to minocycline, such as lupus or connective tissue disease. Patients were considered evaluable if they had a dose limiting toxicity (DLT) during the DLT window or if they completed at least 28 days of minocycline and two infusions of bevacizumab without a DLT. The DLT window included the radiation and 28 days afterward. During dose escalation, unevaluable patients were replaced.

### Interventions

Bevacizumab was given at the standard, FDA-approved dose of 10 mg/kg IV every two weeks. Radiation was individualized based on the best judgment of the radiation oncologist. Most subjects received 3750 centigray (cGy) in 15 fractions or 4000 cGy in 20 fractions. Details of radiation planning are given in Table 2. The gross tumor volume (GTV) was defined mostly as the T1 enhancing tumor, but given the effects on MRI imaging of anti-angiogenic agents the T2/FLAIR abnormality could be included in the GTV at the judgment of the treating radiation oncologist. The median clinical target volume (CTV) margin was 0.5 cm (Range 0–2 cm). The size of the CTV and the decision of whether to include T2/FLAIR in the GTV were based on the judgment of the treating radiation oncologist taking into consideration MRI changes over time, prior radiation, and anatomic location. The median planning treatment volume (PTV) margin was 0.3 cm (Range 0–0.7 cm). The median PTV size was 192.3 cm^3^ (Range 0.3–487 cm^3^). The median RT dose was 3750 cGy (Range 2000–5400 cGy) in 15 fractions (Range 5–30). Intensity modulated radiation therapy (IMRT) was used in all patients but one, who received stereotactic radiosurgery (SRS). Minocycline was taken orally twice a day starting on the day prior to radiation and continuing until progression or intolerance. Three dose levels of minocycline were tested: 100 mg twice a day, 200 mg twice a day, and 400 mg twice a day.

### Safety assessments

The primary endpoint was the rate of adverse events during and up to 28 days after radiation. All patients who received a dose of minocycline were included in the safety analysis. DLTs were defined as grade 3 or intolerable grade 2 toxicities (other than anemia, lymphopenia, high cholesterol, or weight gain) related to the minocycline occurring during or within 28 days of the end of radiation.

### Efficacy and PRO assessments

The secondary endpoints were progression-free survival (PFS) at three (PFS3) and 6 (PFS6) months from the beginning of study treatment, the overall response rate, and changes over time in symptom burden and cognitive function. PFS was defined from the date of start of minocycline until the day of documented disease progression or death. PFS was only assessed in patients on the maximal tolerated dose (MTD) of minocycline. Patients who declined follow-up or did not complete radiation were censored at the time of study withdrawal. Patient symptom burden and symptom interference were measured using the MDASI-BT [28]. Cognitive function was measured using COGSTATE brief battery (detection test (DET), identification test (IDN), one card learning test (OCLT), and Groton Maze Learning Test (GMLT)) [29] Symptom burden and cognitive function were measured at baseline, week 4, week 12, and week 26 before patients were seen by the neuro-oncologist or received any MRI results.

### Ethics

The trial was approved by the University of Utah Institutional Review Board (IRB). It was registered on clinicaltrials.gov, NCT01580969, prior to beginning enrollment.

## Results

### Demographics and clinical characteristics

Twenty-three patients were screened between July, 2012 and November, 2017, of which twenty-two were eligible and enrolled on the trial. Clinical characteristics are summarized in Table 1. Six people enrolled at the 100 mg twice a day dose level, three at the 200 mg twice a day dose level and thirteen at the 400 mg twice a day dose level. All patients have completed follow up and have died. Three people enrolled at the lowest dose did not complete radiation due to patient preference despite not having any DLT, so they were replaced (Table 2).

**Table 1:** Demographics of participants

Characteristic	Number		%
Gender			
Male	14		64
Female	7		32
Declined	1		5
Age			
Median		55.5	
Range		31–76	
Race			
White	20		91
Declined	2		9
Ethnicity			
Non-hispanic	19		86
Hispanic	1		5
Declined	2		9
KPS			
60	5		23
70	1		5
80	11		50
90	5		23
Prior therapies			
Median		3	
Range		2–6	
Time from last surgery (months)			
Median		41	
Range		2.3–153	
Original histology			
Anaplastic astrocytoma	3		14
Grade II astrocytoma	2		9
GBM	13		59
Grade II oligodendroglioma*	1		5
Anaplastic oligodendroglioma	1		5
Anaplastic oligoastrocytoma*	1		5
Grade IV glioneuronal tumor	1		5

^*^The 1p/19q was not codeleted, so by 2016 WHO criteria, these tumors would have been classified as astrocytomas

*KEY: GBM* glioblastoma, *KPS* karnofsky perfromance status

**Table 2:** Radiation parameters

Patient number	Location	GTV	CTV margin (cm)	PTV margin (cm)	PTV volume (cm3)	Type	Number of fractions planned	Total dose planned (cGy)	Number of fractions delivered	Completed radiation
1		Enhancing	2 + FLAIR	0.3	667	IMRT	15	3750	15	Y
2		FLAIR + enhancing	0.5	0.3	487	IMRT	20	4000	20	Y
3		Enhancing	2.5 + FLAIR	0.3	398	IMRT	20	4000	20	Y
4		FLAIR + enhancing	1	0.3	398	IMRT	15	3750	15	Y
5		FLAIR + enhancing	1	0.3	397	IMRT	20	4000	13	N
6		Enhancing	1	0.3	195	IMRT	15	3750	15	Y
7		FLAIR + enhancing	0	0.5	225	IMRT	15	3750	15	Y
8		FLAIR + enhancing	0	0.5	145	IMRT	20	4000	20	Y
9		Enhancing mass	0.5	0	36	IMRT	15	3750	15	Y
10		Enhancing	0.5	0.3	190	IMRT	15	3750	15	Y
11		FLAIR + enhancing	1	0.3	442	VMAT	15	3750	15	Y
12		Enhancing	0	0.3	148	IMRT	10	3500	10	Y
13		Enhancing	0	0.3	181	VMAT	15	3750	15	Y
14		Enhancing	1	0.3	245	IMRT	22	4400	22	Y
15		FLAIR + enhancing	0	0.3	394	IMRT	15	3750	5	N
16		FLAIR + enhancing	0	0.3	216	IMRT	15	3750	15	Y
17		FLAIR + enhancing	1	0.7	443	VMAT	30	5400	5	N
18		FLAIR + enhancing	0	0.3	81.4	IMRT	15	3750	15	Y
19		FLAIR + enhancing	0	0.3	100	VMAT	15	3750	15	Y
20		FLAIR + enhancing	0	0.3	118	VMAT	15	3750	2	N
21*	Splenium	FLAIR + enhancing	0	0.3	34	VMAT	14	3500	9	N
21	Brachium pontis	FLAIR + enhancing	0	0.1	0.3	SRS	5	2000	2	N
22		Enhancing	0.5	0.3	112	VMAT	15	3750	15	Y

*KEY: IMRT* static field intensity modulated radiation therapy, *VMAT* volumetric modulated arc therapy, *SRS* stereotactic radiosurgery, *FLAIR* fluid attenuation inversion recovery

^*^Patient 21 had two separate lesions radiated in two discontiguous fields on trial. All other patients had one contiguous radiation field

Most patients had GBM or multiply recurrent lower grade gliomas without approved systemic treatment options. All patients treated at the 400 mg twice a day dose had either grade III or IV gliomas at initial diagnosis. Of the 22 patients, 13 had unknown IDH status, although one of these was a 1p/19q codeleted anaplastic oligodendroglioma that was presumably IDH mutated and two were GBMs with EGFRvIII mutation that were presumably wild-type. Of the rest, 6 were IDH wild-type, including 4 GBMs, 1 grade IV glioneuronal tumor, and 1 grade II astrocytoma., and three were IDH mutated, including one GBM and two anaplastic astrocytomas. The tumor that was called anaplastic oligodendroglioma histologically was 1p/19q codeleted, but the tumor that was called grade II oligodendroglioma was not 1p/19q codeleted so by current criteria would have been called an astrocytoma.

### Safety

The maximum tolerated dose of minocycline in this population was 400 mg twice a day. Of the patients who started at 400 mg twice a day, nine (70%) were able to complete radiation without stopping or reducing the minocycline. Adverse events were consistent with the known side effect profiles of minocycline, bevacizumab, and radiation. Grade 3 or higher adverse events and adverse events that occurred in more than one person are summarized in Table 3. Grade 3 or higher adverse events occurred in 24% of subjects and all resolved with supportive care or holding the minocycline. No patient had symptomatic radiation necrosis or radiation necrosis requiring treatment. There were no DLTs in the dose escalation and one DLT in the dose expansion (grade 3 nausea, dehydration, and confusion possibly related to the minocycline).

**Table 3:** Adverse events (AEs)

Toxicity code	Dose Level 0: 100 mg	Dose Level 1: 200 mg	Dose Level 2: 400 mg
*Total patients experiencing any G3-4 AE*	0/3	1/3	3/13
Diarrhea	0	0	1
Hypertension	0	0	1
Hypokalemia	0	1	0
Insomnia	0	0	1
Nausea	0	0	1
Thromboembolic event	0	0	2
Urinary incontinence	0	0	1
*Total patients experiencing any G1-2 AE*	3/3	3/3	11/13
Anorexia	0	0	4
Ataxia	0	0	2
Constipation	0	0	3
Cough	0	0	4
Dehydration	0	0	2
Dermatitis radiation	1	0	2
Diarrhea	0	0	4
Dizziness	1	1	6
Dysarthria	0	0	2
Edema limbs	0	0	2
Fatigue	1	1	4
Gait disturbance	1	0	2
Headache	2	2	2
Hoarseness	0	0	2
Hypertension	1	2	1
Insomnia	0	0	2
Memory impairment	0	0	3
Muscle weakness	0	0	3
Nausea	0	1	5
Nervous system disorders—Other	0	1	2
Vomiting	0	1	3

### Efficacy

PFS and OS were assessed in all patients who started treatment at the 400 mg twice a day dose. The PFS3 in this group was 64.6% (95% confidence interval (CI) 42.8–99.8%), and the PFS6 was 27.7% (95% CI 10.6–72.3%), which compares favorably with PFS3 and PFS6 seen in other trials of patients who progressed on bevacizumab. The median PFS was 3.8 months (95% CI 2.5 months–infinity), and the median OS was 6.4 months (95% CI 4.2 months–12.1 months). Radiographic responses were rare, with one person with a gemistocytic astrocytoma treated at the 100 mg twice a day dose level having a complete response and one person with a GBM treated at the 200 mg twice a day level having a partial response. For all 22 people who enrolled on the trial, the median PFS was 3.8 months (95% CI 2.5–8.1 months). The PFS3 was 65.3% (95% CI 47.5–89.8%), and the PFS6 was 35.2%, (95% CI 19.4–63.8%).

### Symptom burden and cognitive outcomes

**Fig. 1:**
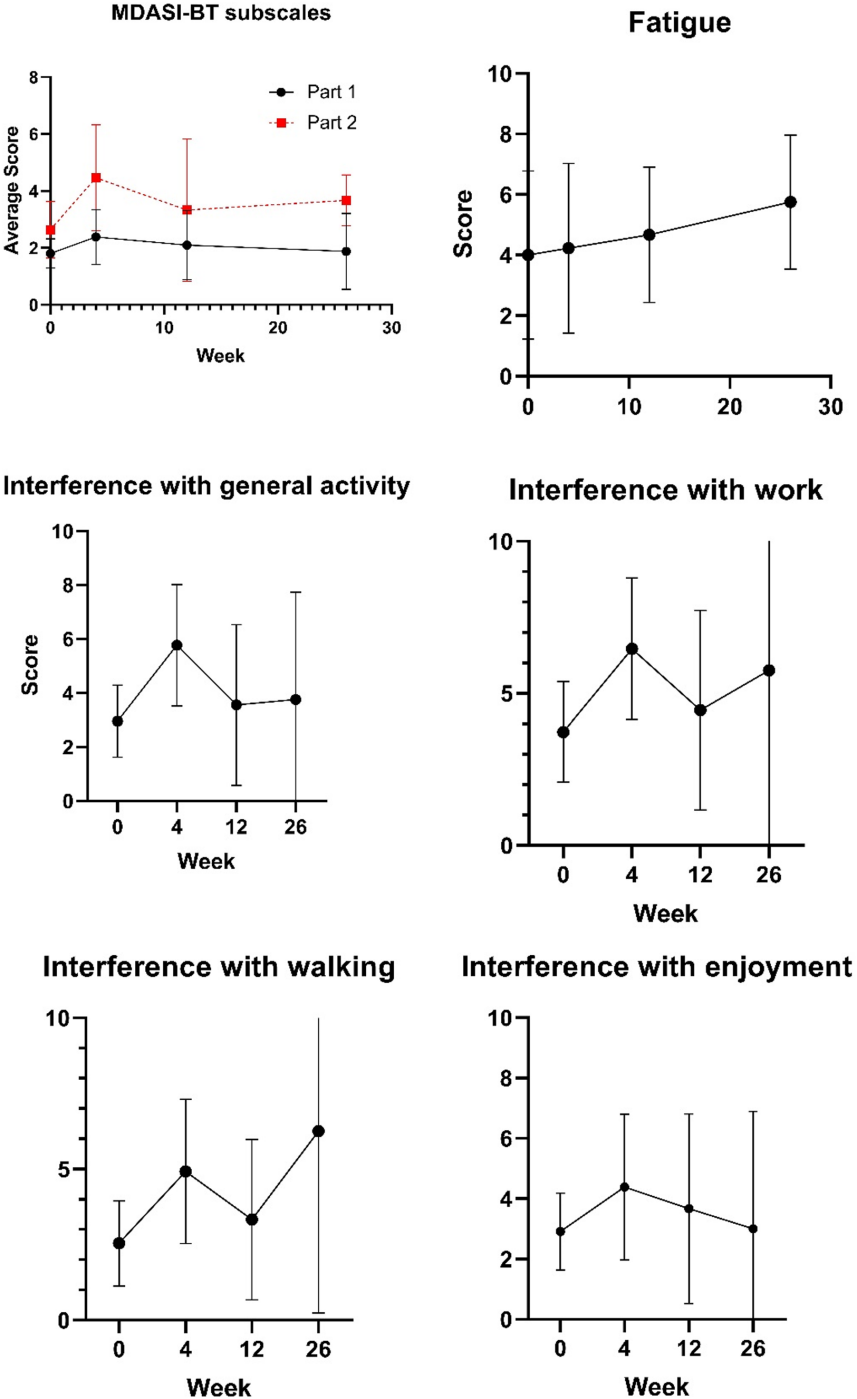
MDASI-BT results for symptom burden and symptom interference subscores (**a**) and for items with moderate or higher average scores (**b**-f). Mean and 95% CI are presented for each time point

Compliance with the MDASI-BT was high with 100% compliance at baseline (22 out of 22 eligible patients), week 12 (nine out of nine eligible patients), and week 26 (four out of four eligible patients) and 93% compliance at week 4 (13 out of 14 eligible patients). At baseline, the average MDASI-BT score was in the moderate range (4–6) for fatigue and memory problems. At week 4, the average MDAS-BT score was in the moderate range for fatigue and memory, and the average interference score was in the moderate range for general activity, walking and enjoyment of life, while the interference with work score had gone into the severe range (> 6). By week 12, however, the interference scores were all below moderate, except for interference with work, which was still in the moderate range. Average scores for the two subscales of the MDASI-BT and for fatigue, interference with general activity, interference with work, interference with walking, and interference with employment are shown in Fig. 1.

Compliance with cognitive testing was 73% at baseline, 69% at week 4, 55% at week 12, and 75% at week 26. Missing test data was almost exclusively due to inability to complete the testing due to slow cognition or difficulty with attention. Thus, the cognitive function of the patients without data on cognitive testing is likely worse than those with testing. Summary scores are shown in Table 4. Of those that completed testing, no significant cognitive declines were seen from baseline, except for the IDN, which declined from an average of 2.79 s to 2.86 s (p = 0.02) from baseline to 4 weeks. The IDN test measures processing speed. In particular, the DET, which is a measurement of attention, OCLT, which is a measurement of working memory, and GMLT, which is a measurement of visual learning and memory, did not worsen for people who did not progress.

**Table 4:** Cognitive testing results. COGSTATE results for the detection test (DET), identification test (IDN), one card learning test (OCLT), and Groton Maze Learning Test (GMLT)

Cognitive function descriptive statistics			
Question	n completed	Mean (Seconds)	SD
Baseline cognitive function			
DET	14	2.63	0.16
IDN	16	2.79	0.09
OCLT	16	0.89	0.17
GMLT	16	89.31	30.81
4 Week cognitive function			
DET	9	2.67	0.15
IDN	8	2.86	0.09
OCLT	9	0.81	0.09
GMLT	9	75.33	27.45
12 Week cognitive function			
DET	5	2.65	0.14
IDN	5	2.79	0.11
OCLT	5	0.82	0.05
GMLT	5	77.40	28.02
26 Week cognitive function			
DET	3	2.79	0.11
IDN	3	2.88	0.09
OCLT	3	0.87	0.14
GMLT	3	65.33	19.86

## Discussion

Our trial demonstrates the safety of reirradiation with bevacizumab and minocycline at a starting dose of 400 mg twice a day for recurrent GBM after progression on radiation, temozolomide, and bevacizumab. The trial accrued before the approval of tumor treating fields. It adds a prospective trial to the literature showing that reirradiation of high grade gliomas after bevacizumab failure can be performed with acceptable tolerability.

The only other published studies of reirradiation after progression on bevacizumab for patients with high grade glioma included retrospective analyses by Schernberg et al. (n = 13) [19] and Shi et al. (n = 30) [20] Although the median overall survival in those series (5.4 months and 4.8 months, respectively) was only slightly lower than ours (6.4 months), radiation doses were different and more radiation was performed stereotactically. In addition, the median PTV in our trial is about twice that in the Schernberg series and about eight times that in the Shi series. The retrospective nature of those series also could introduce selection bias. Thus, the survival in this prospective trial compares favorably to those series. Moreover, the one prospective trial of reirradiation in recurrent GBM for bevacizumab-naïve patients, Radiation Therapy Oncology Group (RTOG) 1205, failed to show an incremental benefit of radiation given via 35 Gy in 10 fractions when added to bevacizumab in this population [30].

Trials of systemic therapies in gliomas after progression on bevacizumab have been disappointing. For example, veribulin had a PFS1 of 20% and nintedanib has a PFS3 of 0%, compared to the PFS3 of 65% in this trial [31, 32]. Tumor treating fields, which have gone on to FDA approval and a successful phase 3 trial in upfront treatment of GBM, gave a median OS of 6 months in patients with prior bevacizumab failure in a post-hoc subgroup analysis of the EF-11 trial [3]. Although cross-trial comparison has known pitfalls, the OS curve for tumor treating fields is similar to that seen with minocycline, bevacizumab, and radiation in our trial. Thus, prospective evaluation of the benefit of minocycline is warranted.

The symptom burden and cognitive data support the tolerability of this regimen. Patients and providers might fear that additional treatments, particularly reirradiation, may prolong life at the expense of quality of life anticipating significant declines in symptoms or cognition. In this trial, the overall symptom burden and cognitive function of survivors remained stable without clinically significant changes before tumor progression. The interference of symptoms with function and quality of life did increase immediately after radiation but then returned back toward baseline over time. No other trial of reirradiation has assessed quality of life, symptom burden, or neurocognitive function systematically.

The main limitation in this trial is the inability to separate out the effects of the radiation, bevacizumab, and the minocycline. In addition to the usual difficulties with cross trial comparisons of single-arm, single-center trials, there are few other studies of radiation in bevacizumab-refractory GBM with which to compare. Survival in a small, single-arm, single institution trial such as this may not generalize. The OS in this trial does not seem markedly longer than radiation alone or tumor treating fields, suggesting further breakthroughs are needed in this population. In addition, radiation parameters in this trial were somewhat heterogeneous, as the radiation prescription was based on the individualized judgment of the radiation oncologist. However, we think this variability increases the generalizability of our results to real world practice. Lastly, tissue from immediately prior to treatment was not available, so no correlative molecular studies were performed to identify either the molecular subtypes of the participants’ tumors or predictors of response.

This trial demonstrates the safety and tolerability of minocycline, reirradiation, and bevacizumab. A recently completed Phase I trial at the University of Utah is assessing the safety and efficacy of minocycline added to radiation and temozolomide in the upfront treatment of high grade gliomas. (NCT02272270). This upfront trial includes correlative tissue and radiomic studies not possible in the recurrent setting, which will help inform the optimal population for future randomized trials. Future studies comparing radiation and bevacizumab with and without minocycline are warranted.

## Conclusion

Reirradiation with bevacizumab and minocycline is safe in bevacizumab-refractory high grade gliomas. Further studies with minocycline or other radiosensitizers are indicated.
